# Supplementary material for: Paraoxonase 2 (PON2) Deficiency Reproduces Lipid Alterations of Diabetic and Inflammatory Glomerular Disease and Affects TRPC6 Signaling
Source: Cells. 2022 Nov 16;11(22):3625. doi: 10.3390/cells11223625 (PMC9688324; doi:10.3390/cells11223625)
Supplement: Supplementary file 1 [file cells-11-03625-s001.zip › cells-1986621-supplementary.pdf]

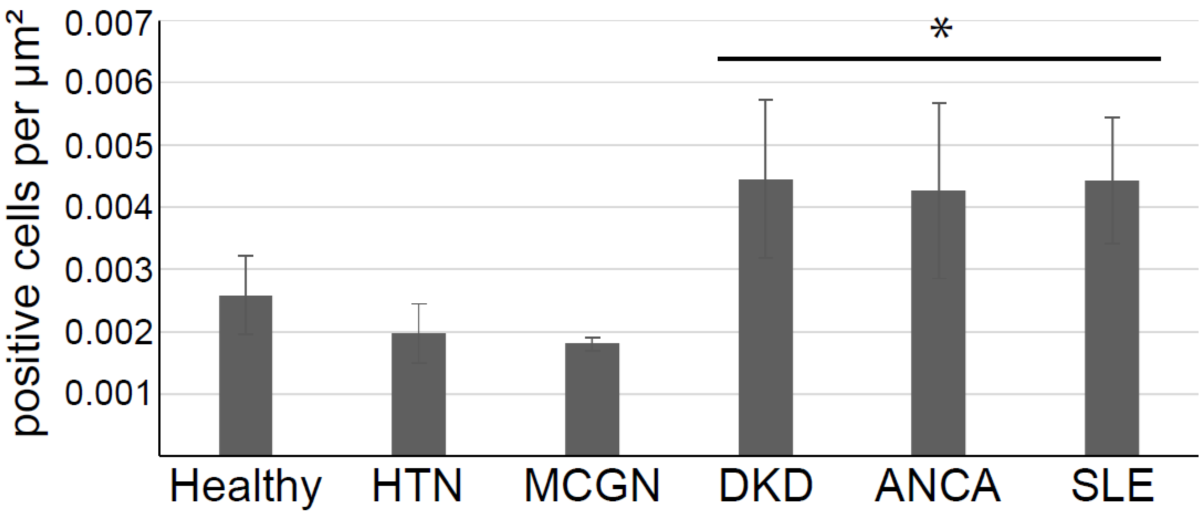

Suppl. Figure S1:

PON2-positive cells quantified per glomerular area. More PON2 positive cells are found in DKD and inflammatory glomerular disease; $p<0.05$. (Number of patients/glomeruli examined: Healthy n2/4; HTN 2/5; MCGN 2/2; DKD 5/10; ANCA 3/6; SLE 2/2).

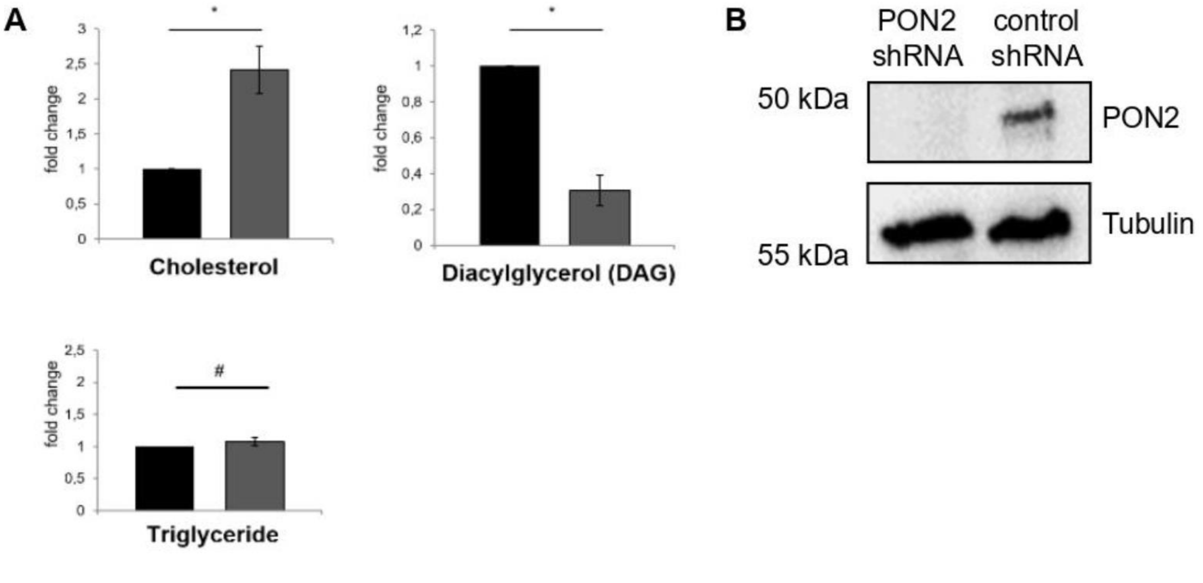

Suppl. Figure S2:

(A) Thin layer chromatography analysis of cholesterol, triglyceride and DAG content in cultured murine podocytes. Relative lipid content in PON2-deficient podocytes (grey bars) is shown as fold change from control podocytes (black bars) (cholesterol 2.42 fold, SD 0.34; DAG 0.31 fold, SD 0.09; trigl.1.07 fold, SD 0.07). *$p<0.05$; #=not significant. (B) Immunoblot analysis of lysates of stable murine podocyte cell lines expressing PON2-specific shRNA (PON2 shRNA) or scrambled shRNA (control shRNA). Cells expressing PON2 shRNA show efficient knockdown of PON2 on protein level.

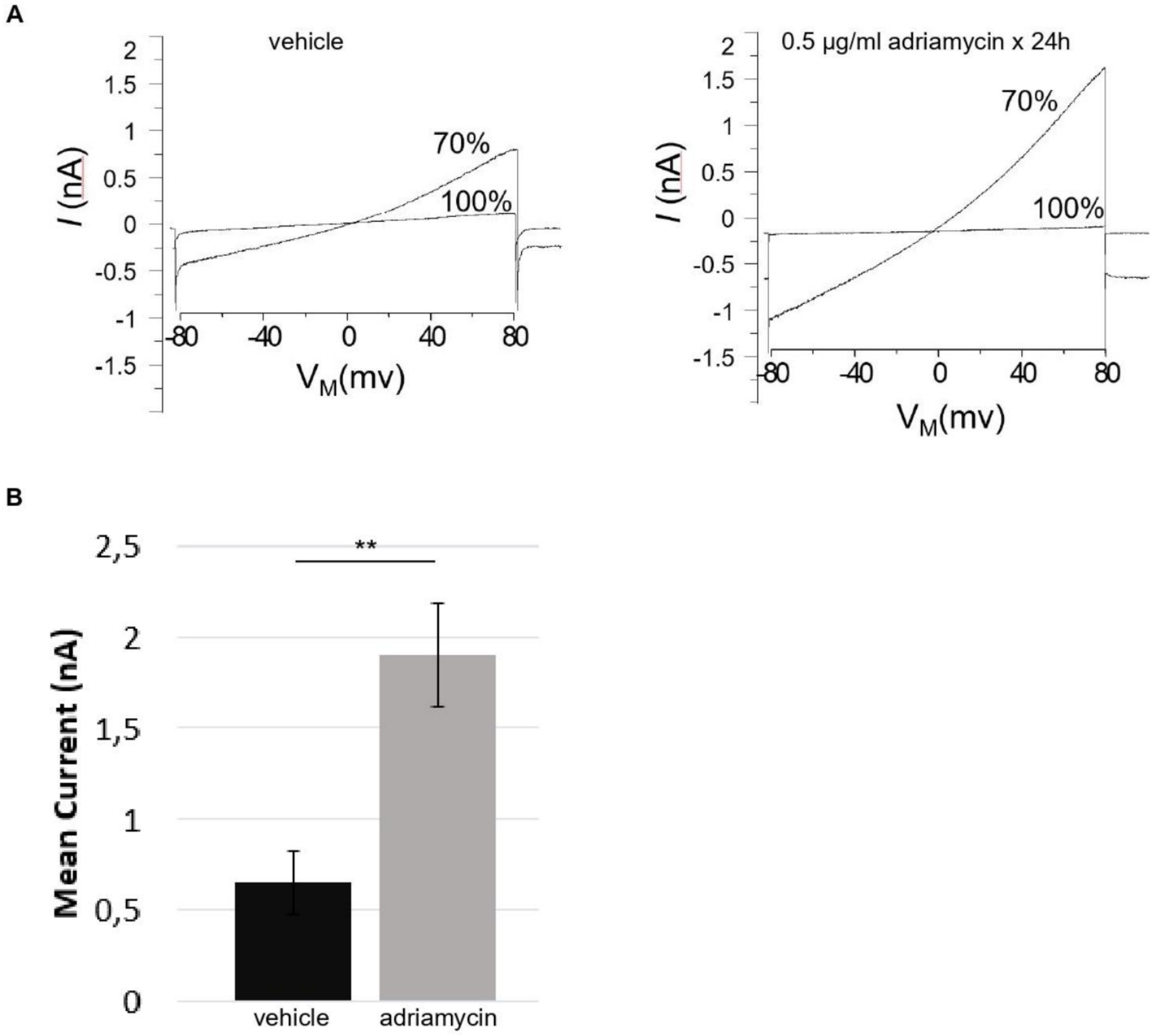

Suppl. Figure S3:

(A) After treatment of murine wildtype podocytes with vehicle or adriamycin, whole cell voltage clamp recordings showed increased TRPC6 conductance in cells treated with adriamycin (mean 0.66 nA, SD 0.19 vs. 1.90 nA, SD 0.29; n=10 cells each condition). (B) Mean currents were significantly higher in adriamycin-treated podocytes as compared to control (*$p<0.05$).

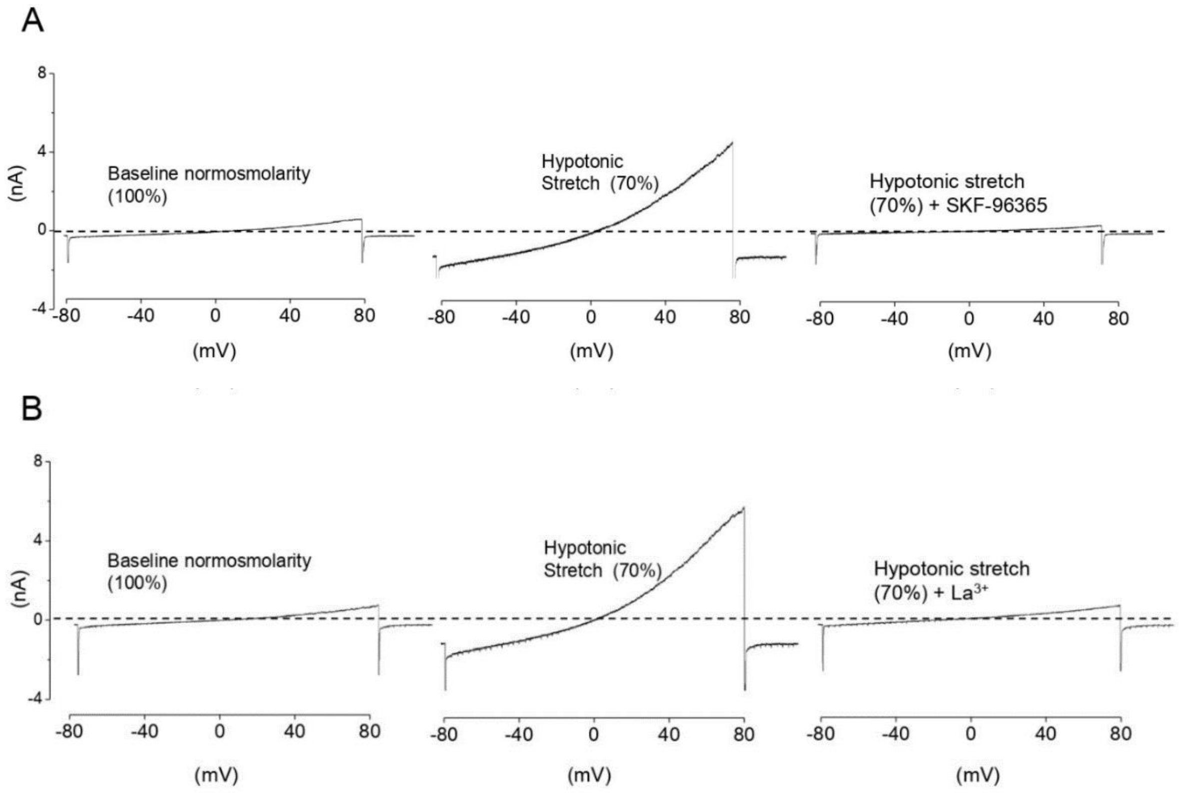

Suppl. Figure S4:

Stretch-induced TRPC6 conductance in PON2 deficient podocytes was blocked by specific TRPC6 inhibitors SKF-96365 (A) and Lanthanum (B).

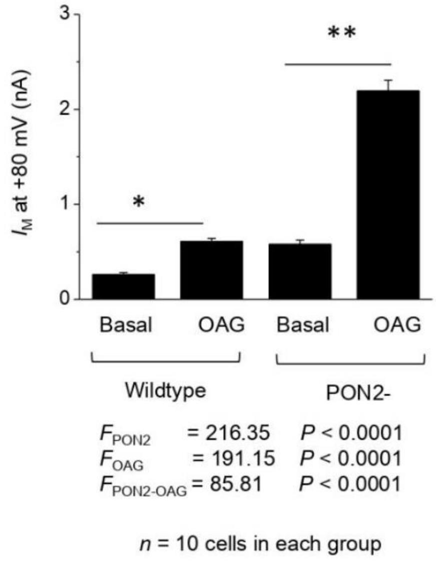

Suppl. Figure S5:

(A) PON2-deficient podocytes showed increased TRPC6 conductance in ramp experiments at baseline and a massive increase in TRPC6 currents after stimulation with 100 µM OAG. Two-way ANOVA showed significant differences of the independent variables PON2 expression and OAG treatment. In addition, a strong interaction effect was detected

(*p<0.05; **p>0.01) (means: ctr. basal 0.29 nA, SD 0.02; ctr. +OAG 0.61 nA, SD0.05; PON2- basal 0.60, SD 0.07; PON2- +OAG 2.25 nA, SD 0.12).

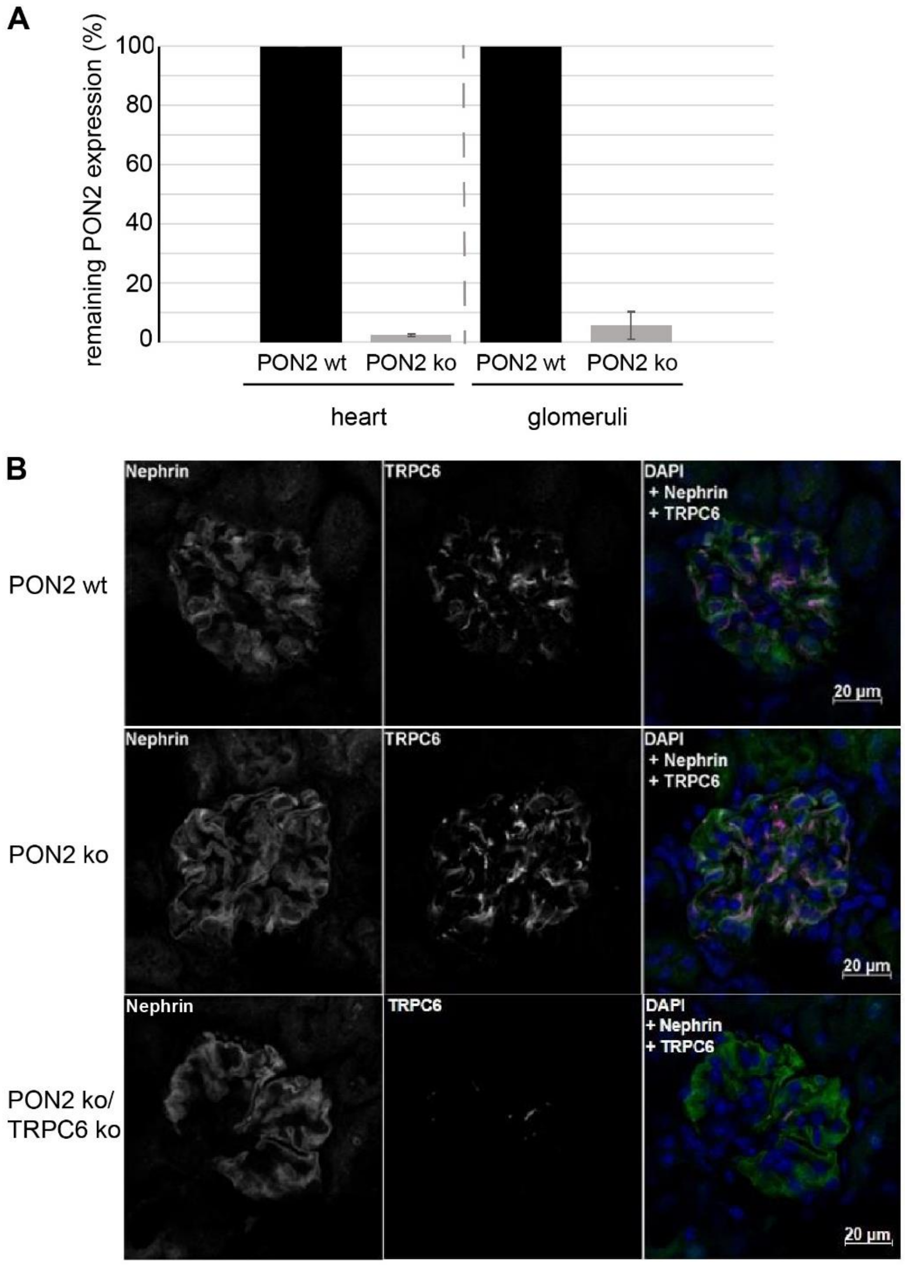

Suppl. Figure S6:

(A) Validation of PON2 knockout was performed by quantitative PCR for PON2 on heart tissue and isolated glomeruli of PON2 wt and PON2 ko mice. Remaining PON2 expression was 2.38% in heart tissue and 5.69% in glomeruli. (B) Knockout of TRPC6 was substantiated by immunofluore1scence staining of kidney sections of mice with indicated genotypes.
